# Supplementary material for: Enhanced HIV-1 Neutralizing Antibody Breadth in HTLV-2 Co-Infected Individuals: Influence of Antiretroviral Regimen and B Cell Subset Distribution
Source: Vaccines (Basel). 2025 Jun 13;13(6):639. doi: 10.3390/vaccines13060639 (PMC12197736; doi:10.3390/vaccines13060639)
Supplement: Supplementary file 1 [file vaccines-13-00639-s001.zip › vaccines-3655987-supplementary.pdf]

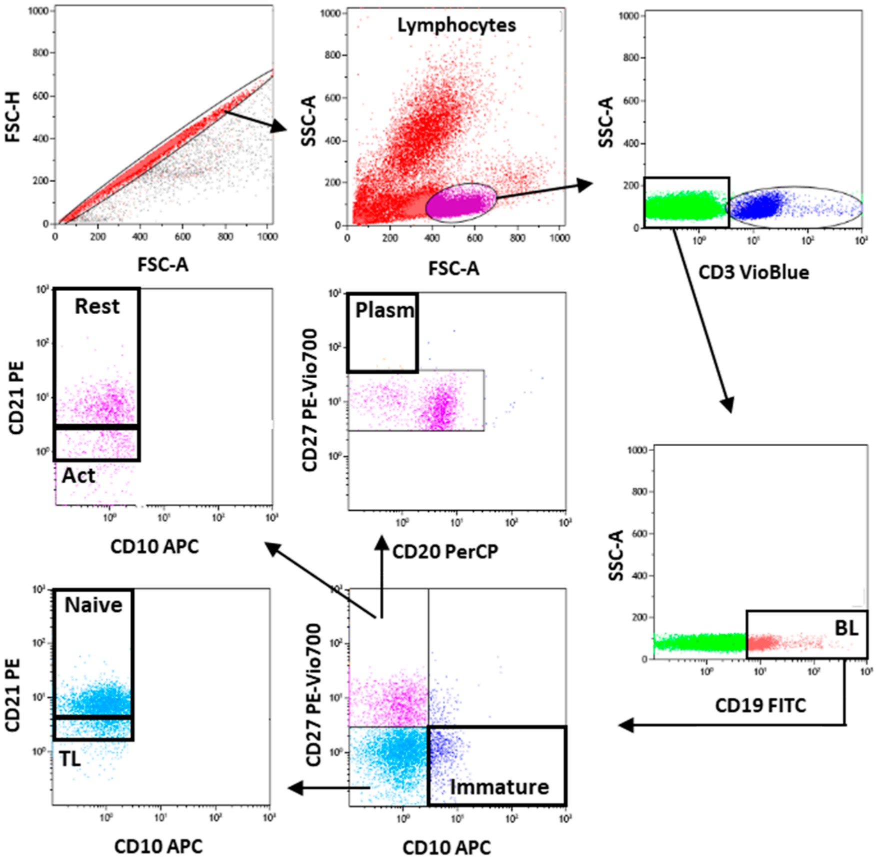


**Supplementary Figure S1. Flow-cytometric gating strategy for B-cell subpopulation analysis for representative sample. After singlet cells were gated (FSS-A/FSS-H dot plot), positive cells for live/dead and anti-CD3 were excluded. B lymphocytes (BL, CD3$^-$ CD19$^+$) were ploted according to the expression of CD10 and CD27. Six B-cell subpopulations were further defined as: immature/transitional (CD10$^+$ CD27$^-$), naïve (CD10$^-$ CD27$^-$ CD21$^{hi}$), tissue-like memory (TL, CD10- CD27- CD21$^{lo}$), resting memory (Rest, CD10$^-$ CD27$^+$ CD21$^{hi}$), activated memory (Act, CD10$^-$ CD27$^+$ CD21$^{lo}$), and plasmablasts (Plasm, CD10$^-$ CD27$^{++}$ CD20-CD21$^{lo}$. FSC-A, forward scatter-area; SSC-A, side scatter-area; FSC-H, forward scatter-height.**
